# Additive Intraocular Pressure-Lowering Effects of Ripasudil with Glaucoma Therapeutic Agents in Rabbits and Monkeys

**DOI:** 10.1155/2017/7079645

**Published:** 2017-04-28

**Authors:** Yoshio Kaneko, Masayuki Ohta, Tomoyuki Isobe, Yuto Nakamura, Ken Mizuno

**Affiliations:** Tokyo New Drug Research Laboratories, Kowa Co. Ltd., 2-17-43 Noguchicho, Higashimurayama, Tokyo 189-0022, Japan

## Abstract

Ripasudil hydrochloride hydrate (K-115), a specific Rho-associated coiled-coil containing protein kinase (ROCK) inhibitor, is developed for the treatment of glaucoma and ocular hypertension. Topical administration of ripasudil decreases intraocular pressure (IOP) by increasing conventional outflow through the trabeculae to Schlemm's canal, which is different from existing agents that suppress aqueous humor production or promote uveoscleral outflow. In this study, we demonstrated that ripasudil significantly lowered IOP in combined regimens with other glaucoma therapeutic agents in rabbits and monkeys. Ripasudil showed additional effects on maximum IOP lowering or prolonged the duration of IOP-lowering effects with combined administration of timolol, nipradilol, brimonidine, brinzolamide, latanoprost, latanoprost/timolol fixed combination, and dorzolamide/timolol fixed combination. These results indicate that facilitation of conventional outflow by ripasudil provides additive IOP-lowering effect with other classes of antiglaucoma agents. Ripasudil is expected to have substantial utility in combined regimens with existing agents for glaucoma treatment.

## 1. Introduction

Rho-kinase (Rho-associated coiled-coil containing protein kinase; ROCK), a member of the serine-threonine protein kinases, is an effector protein of low-molecular-weight G-protein, Rho [1]. ROCK has two isoforms, ROCK-1 and ROCK-2, which are extensively distributed throughout the various organs, including the ocular tissues [2, 3]. ROCK binds with Rho to form a Rho/ROCK complex and regulates various physiological functions, such as smooth muscle contraction, chemotaxis, neural growth, and gene expression [1, 4–8]. However, aberrant regulation of ROCK levels in the eyes has been shown to be involved in the pathogenesis of diabetic retinopathy, age-related macular edema, cataract, corneal dysfunction, retinal disorders, and glaucoma [9–20].

Glaucoma is primarily a disease affecting the optic nerve head that characteristically leads to visual field loss and ultimately blindness. Primary open-angle glaucoma (POAG), the commonest form of glaucoma, often observed chronic elevation of intraocular pressure (IOP). These were developed as a result of pathologically increased resistance to the drainage of the aqueous humor through outflow pathways [21]. IOP reduction is currently the only reliable and evidence-based management approach for the treatment of glaucoma [22]. The strategies of glaucoma treatment are decided according to the stages of glaucoma, types, and different conditions, with pharmacological agents considering the first-line therapy in most types of glaucoma [23]. The ocular hypotensive mechanisms of currently available antiglaucoma agents are categorized into two types. One is to promote uveoscleral outflow, such as prostaglandin (PG) analogs, *αβ*-adrenergic receptor blockers, *α*1-adrenergic receptor blockers, and *α*2-adrenergic receptor agonists, and the other is to suppress aqueous humor production, such as *β*-adrenergic receptor blockers, carbonic anhydrase inhibitors (CAI), and *αβ*-adrenergic receptor blockers [23]. However, reduction of IOP below the target level is often challenging with monotherapy [24]. Consequently, there is a great clinical need for a novel class of agents, which possesses potent IOP-lowering effects, and can be used with other agents for combination therapy.

Ripasudil is the first-in-class ROCK inhibitor ophthalmic agent developed for the treatment of glaucoma and ocular hypertension [25–32]. In the previous study, we showed that ripasudil decreased IOP by potentiation of the outflow facility from the conventional outflow route [26, 27]. The mechanism of actions of ripasudil is different from that of other agents, such as promotion of uveoscleral outflow and suppression of aqueous humor production. In this study, we demonstrated that topical instillation of ripasudil ophthalmic solution with other glaucoma therapeutic agents, such as *β*-blocker, *αβ*-blocker, *α*2-agonist, CAI, and PG analogs, further reduced IOP and for a longer duration.

## 2. Materials and Methods

### 2.1. Animals

Male Japanese white rabbits weighing 2.0–3.0 kg and male cynomolgus monkeys weighing 5.0–8.5 kg (5 years or older) were used in this study. The rabbits were housed in an air-conditioned room (22–25°C, 50%–70% humidity) lit from 7:00 to 19:00, and they were allowed food and water ad libitum throughout the experiments. The monkeys were housed in an air-conditioned room (22–28°C, 40%–80% humidity) lit from 7:00 to 19:00, and they were provided 100 g of feed daily between 9:00 and 12:00, with the exception that feed were provided after the last measurement on each day of IOP measurement. All studies were conducted in accordance with the ARVO Statement for the Use of Animals in Ophthalmic and Vision Research and were approved by the Animal Ethics Committee of Kowa Tokyo New Drug Research Laboratories.

### 2.2. Chemicals and Drug Preparation

Ripasudil was synthesized at Tokyo New Drug Research Laboratories, Kowa Co. Ltd. (Tokyo, Japan) and was dissolved in a vehicle containing preservative for clinical use as an ophthalmic agent. 0.25% nipradilol (HYPADIL Kowa ophthalmic solution 0.25%) was purchased from Kowa Pharmaceutical Co. Ltd. (Tokyo, Japan); 1% brinzolamide (Azopt 1% ophthalmic suspension) was purchased from Alcon Japan Ltd. (Tokyo, Japan); 0.1% brimonidine (Aiphagan ophthalmic solution 0.1%) was purchased from Senju Pharmaceutical Co. Ltd. (Osaka, Japan); 0.005% latanoprost (Xalatan eye drops 0.005%) and 0.005% latanoprost/0.5% timolol fixed combination (Xalacom combination eye drops) were purchased from Pfizer Inc. (Tokyo, Japan); and 0.5% timolol (Timoptol Ophthalmic Solution 0.5%) and 1% dorzolamide/0.5% timolol fixed combination (COSOPT ophthalmic solution) were purchased from Santen Pharmaceutical Co. Ltd. (Osaka, Japan).

### 2.3. Method of Topical Administration

In rabbit experiments, 50 *μ*L of agents were instilled into one eye. For combined regimens with other agents, 0.5% timolol, 0.25% nipradilol, 0.1% brimonidine, or 1% brinzolamide was administrated 5 min after the instillation of 0.4% ripasudil. The contralateral eye was not treated. In monkey experiments, 20 *μ*L of agents was instilled into one eye. For combined regimens with other agents, 0.005% latanoprost (alone) and 0.005% latanoprost/0.5% timolol or 1% dorzolamide/0.5% timolol (in combination) were administrated 5 min after the instillation of 0.4% ripasudil or vehicle. The contralateral eye was not treated.

### 2.4. Measurement of Intraocular Pressure

Pneumotonometers (Model 30 Classic Pneumotonometer; Medtronic Solan Ophthalmic Products Inc., Jacksonville, FL) were used to monitor IOP. For IOP measurements, the eyes were anesthetized by topical instillation of 0.4% oxybuprocaine (0.4% Benoxil ophthalmic solution; Santen Pharmaceutical Co. Ltd., Osaka, Japan). IOP was measured in both eyes prior to the instillation of agents and 0.5, 1, 2, 3, 4, and 5 h after instillation in the albino rabbits. For the experiments with monkeys, IOP was measured before and 1, 2, 4, and 6 h or 1, 2, 3, 4, 6, and 8 h after instillation of agents.

### 2.5. Statistical Analyses

Difference in IOP (⊿IOP) between pretreatment (0 h) and at each time point after the instillation of agents was calculated. All data are expressed as means ± SEs. For ⊿IOP, Tukey's multiple comparison test (comparing means of more than two groups) or Student's *t*-test (comparing means of two groups) was performed at each time point for each treatment group. *P* < 0.05 was predetermined as the criterion of statistical significance for all the analyses.

## 3. Results

### 3.1. Additive IOP-Lowering Effect of Ripasudil with Timolol

**Figure 1:**
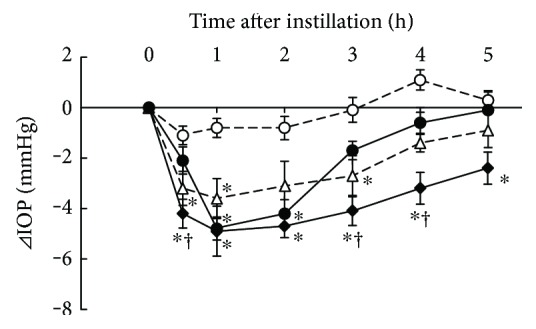
Additive IOP-lowering effect of ripasudil with timolol. Male albino rabbits were administered 50 *μ*L of vehicle (○), 0.4% ripasudil (●), 0.5% timolol (△), or 0.4% ripasudil + 0.5% timolol (◆) into one eye (*n* = 9). The contralateral eye was not treated. IOP were measured using pneumotonometers prior to the experiments and 0.5, 1, 2, 3, 4, and 5 h after instillation. For combined use of ophthalmic agents, 0.5% timolol was administered 5 min after instillation of 0.4% ripasudil. All data are presented as means ± SEs. ^∗^^,†^*P* < 0.05, compared with vehicle and 0.4% ripasudil, respectively (Tukey's multiple comparison test).

IOP-lowering effects of 0.4% ripasudil, 0.5% timolol, and combined treatment of 0.4% ripasudil with 0.5% timolol were demonstrated in rabbits (Figure 1). Compared with vehicle, ripasudil significantly lowered the IOP 1 and 2 h after instillation, and timolol significantly lowered 0.5, 1, and 3 h after instillation. Combined treatment of ripasudil and timolol significantly lowered IOP at 0.5, 1, 2, 3, 4, and 5 h after instillation compared with vehicle and at 0.5, 3, and 4 h after instillation compared with ripasudil.

### 3.2. Additive IOP-Lowering Effect of Ripasudil with Nipradilol

**Figure 2:**
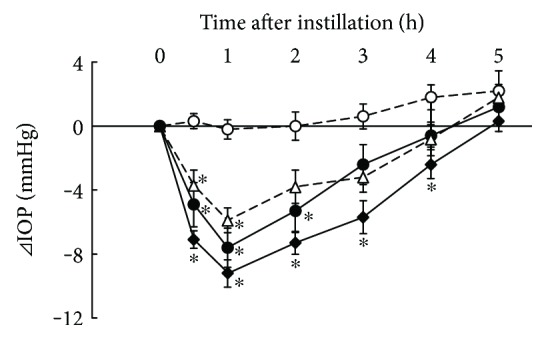
Additive IOP-lowering effect of ripasudil with nipradilol. Rabbits were administered vehicle (○), 0.4% ripasudil (●), 0.25% nipradilol (△), or 0.4% ripasudil + 0.25% nipradilol (◆) into one eye (*n* = 10). IOP were measured 0.5, 1, 2, 3, 4, and 5 h after instillation. For combined use of ophthalmic agents, 0.25% nipradilol was administered 5 min after instillation of 0.4% ripasudil. All data are presented as means ± SEs. ^∗^*P* < 0.05, compared with vehicle (Tukey's multiple comparison test).

IOP-lowering effects of 0.4% ripasudil, 0.25% nipradilol, and combined treatment of 0.4% ripasudil with 0.25% nipradilol were demonstrated in rabbits (Figure 2). Compared with vehicle, a significant IOP-lowering effect was observed at 0.5, 1, and 2 h after instillation of ripasudil; 0.5 and 1 h after instillation of nipradilol; and 0.5, 1, 2, 3, and 4 h after instillation of combined treatment of ripasudil and nipradilol.

### 3.3. Additive IOP-Lowering Effect of Ripasudil with Brinzolamide

**Figure 3:**
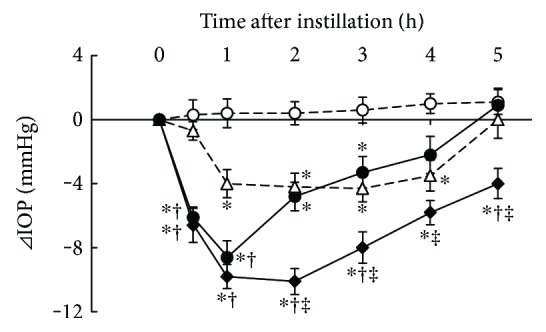
Additive IOP-lowering effect of ripasudil with brinzolamide. Rabbits were administered vehicle (○), 0.4% ripasudil (●), 1% brinzolamide (△), or 0.4% ripasudil + 1% brinzolamide (◆) into one eye (*n* = 10). IOP were measured 0.5, 1, 2, 3, 4, and 5 h after instillation. For combined use of ophthalmic agents, 1% brinzolamide was administered 5 min after instillation of 0.4% ripasudil. All data are presented as means ± SEs. ^∗^^,†,‡^*P* < 0.05, compared with vehicle, 0.4% ripasudil, and 1% brinzolamide, respectively (Tukey's multiple comparison test).

IOP-lowering effects of 0.4% ripasudil, 1% brinzolamide, and combined treatment of 0.4% ripasudil with 1% brinzolamide were demonstrated in rabbits (Figure 3). Compared with vehicle, a significant IOP-lowering effect was observed at 0.5, 1, 2, and 3 h after instillation of 0.4% ripasudil; 1, 2, 3, and 4 h after instillation of brinzolamide; and 0.5, 1, 2, 3, 4, and 5 h after instillation of combined treatment of ripasudil and brinzolamide. Moreover, combination of ripasudil and brinzolamide showed significant IOP-lowering effect at 2, 3, and 5 h against both single instillation of 0.4% ripasudil and 1% brinzolamide.

### 3.4. Additive IOP-Lowering Effect of Ripasudil with Brimonidine

**Figure 4:**
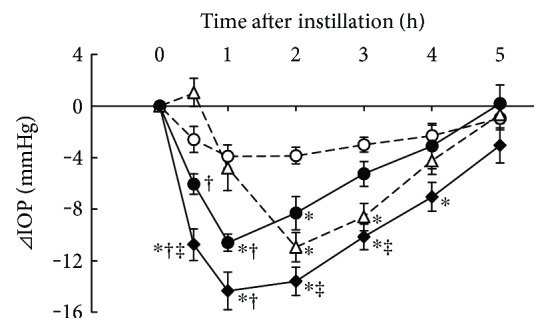
Additive IOP-lowering effect of ripasudil with brimonidine. Rabbits were administered vehicle (○), 0.4% ripasudil (●), 0.1% brimonidine (△), or 0.4% ripasudil + 0.1% brimonidine (◆) into one eye (*n* = 10). IOP were measured 0.5, 1, 2, 3, 4, and 5 h after instillation. For combined use of ophthalmic agents, 0.1% brimonidine was administered 5 min after instillation of 0.4% ripasudil. All data are presented as means ± SEs. ^∗^^,†,‡^*P* < 0.05, compared with vehicle, 0.4% ripasudil, and 0.1% brimonidine, respectively (Tukey's multiple comparison test).

IOP-lowering effects of 0.4% ripasudil, 0.1% brimonidine, and combined treatment of 0.4% ripasudil with 0.1% brimonidine were demonstrated in rabbits (Figure 4). Compared with vehicle, ripasudil significantly lowered the IOP at 1 and 2 h after instillation; 0.1% brimonidine at 2 and 3 h after instillation; and combined treatment of ripasudil and brimonidine at 0.5, 1, 2, 3, and 4 h after instillation. Additionally, combined treatment of ripasudil and brimonidine significantly lowered IOP compared with ripasudil, and brimonidine alone, at 0.5 h after instillation.

### 3.5. Additive IOP-Lowering Effect of Ripasudil with Latanoprost in Cynomolgus Monkeys

**Figure 5:**
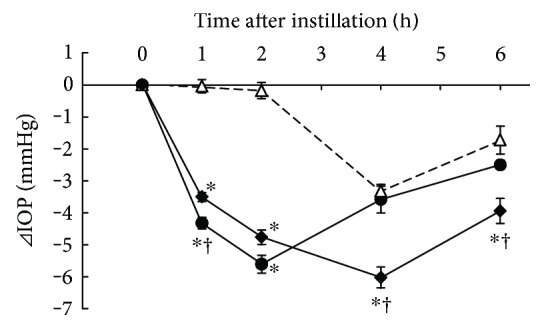
Additive IOP-lowering effect of ripasudil with latanoprost. Male cynomolgus monkeys were administered 20 *μ*L of 0.4% ripasudil (●), 0.005% latanoprost (△), or 0.4% ripasudil + 0.005% latanoprost (◆) into one eye (*n* = 4). The contralateral eye was not treated. IOP were measured using pneumotonometers prior to the experiments and 1, 2, 4, and 6 h after instillation. For combined use of ophthalmic agents, 0.005% latanoprost was administered 5 min after instillation of 0.4% ripasudil. All data are presented as means ± SEs. ^∗^^,†^*P* < 0.05, compared with 0.005% latanoprost and ripasudil, respectively (Tukey's multiple comparison test).

IOP-lowering effects of 0.4% ripasudil, 0.005% latanoprost, and combined treatment of 0.4% ripasudil with 0.005% latanoprost were demonstrated in monkeys (Figure 5). Compared with both single instillation of ripasudil and latanoprost, a significant IOP-lowering effect was observed 4 and 6 h after instillation of the combined treatment of ripasudil and latanoprost.

### 3.6. Additive IOP-Lowering Effect of Ripasudil with Fixed Combination Agents in Cynomolgus Monkeys

**Figure 6:**
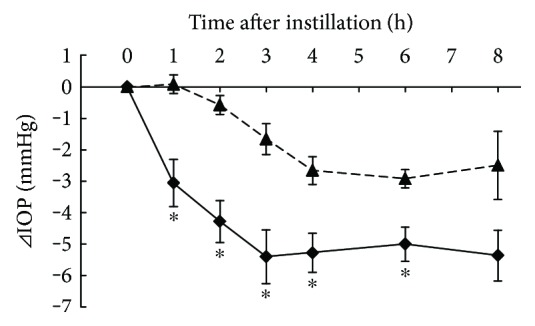
Additive IOP-lowering effect of ripasudil with latanoprost/timolol fixed combination. Monkeys were administered vehicle + 0.005% latanoprost/0.5% timolol fixed combination (▲), or 0.4% ripasudil + 0.005% latanoprost/0.5% timolol fixed combination (◆) into one eye (*n* = 5). IOP were measured before and 1, 2, 3, 4, 6, and 8 h after instillation. 0.005% latanoprost/0.5% timolol fixed combination was administered 5 min after instillation of vehicle or 0.4% ripasudil. All data are presented as means ± SEs. ^∗^*P* < 0.05, compared with vehicle + 0.005% latanoprost/0.5% timolol fixed combination (Student's *t*-test).

**Figure 7:**
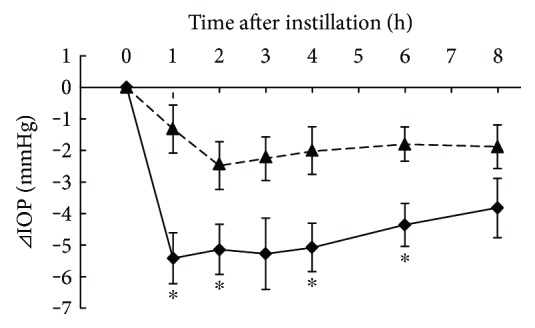
Additive IOP-lowering effect of ripasudil with dorzolamide/timolol fixed combination. Monkeys were administered vehicle + 1% dorzolamide/0.5% timolol fixed combination (▲) or 0.4% ripasudil + 1% dorzolamide/0.5% timolol fixed combination (◆) into one eye (*n* = 5). IOP were measured before and 1, 2, 3, 4, 6, and 8 h after instillation. 1% dorzolamide/0.5% timolol fixed combination was administered 5 min after instillation of vehicle or 0.4% ripasudil. All data are presented as means ± SEs. ^∗^*P* < 0.05, compared with vehicle + 1% dorzolamide/0.5% timolol (Student's *t*-test).

Additive IOP-lowering effects of 0.4% ripasudil with fixed combination (0.005% latanoprost/0.5% timolol or 1% dorzolamide/0.5% timolol) were demonstrated in monkeys. Compared with latanoprost/timolol and vehicle, combination of latanoprost/timolol and ripasudil significantly lowered the IOP 1, 2, 3, 4, and 6 h after administration (Figure 6). Compared with dorzolamide/timolol and vehicle, combination of dorzolamide/timolol and ripasudil significantly lowered the IOP 1, 2, 4, and 6 h after administration (Figure 7).

## 4. Discussion

In this study, we demonstrated the additive IOP-lowering effects of ripasudil topical instillation with other glaucoma therapeutic agents, *β*-blocker, *αβ*-blocker, *α*2-agonist, CAI, PG analogs, and fixed combination. A lot of therapeutic agents are used to manage IOP for glaucoma treatment. For example, PG analogs, *αβ*-blockers, *α*1-blockers, and *α*2-agonists are currently used to promote uveoscleral outflow, and *β*-blockers, CAI, and *αβ*-blockers are used to suppress aqueous humor production. In addition, these agents are used in different ways for glaucoma treatment, such as combined administration of agents, fixed-dose combination formulations, and appropriate agents are selected according to the target IOP of each patient. However, there are unmet medical needs in the market for developing novel class of ocular hypotensive agents, as present antiglaucoma agents are insufficient for obtaining the required reduction of IOP. In this study, we aimed to evaluate the additive IOP-lowering effects of combined regimens of ripasudil and other antiglaucoma agents in rabbits and monkeys. We believe that the mechanism of facilitation via conventional outflow by ripasudil differs from those of other agents.

The pharmacological features of ripasudil have previously been investigated. Ripasudil inhibited both human ROCK-1 and ROCK-2 with IC_50_ values of 0.051 and 0.019 *μ*mol/L, respectively. The inhibitory effect of ripasudil was more potent than that of Y-27632 or fasudil [26]. Inhibitory activities (as in IC_50_ values) of ripasudil on other serine/threonine kinases are approximately 1000-fold less potent than ROCK inhibition. Moreover, ripasudil does not inhibit carbonic anhydrase and has no binding affinity for *α*-, *β*-, and prostanoid receptors. These results indicate that ripasudil is a selective ROCK inhibitor.

In in vivo studies using rabbits and monkeys with normal IOP, a clinical dose of 0.4% ripasudil showed a significant IOP-lowering effect, which was comparable with existing glaucoma therapeutic agents [26, 27]. In a study of aqueous humor dynamics in rabbits, instillation of 0.4% ripasudil significantly increased outflow facility; however, it had no effect on uveoscleral outflow or aqueous flow rate [26]. In in vitro studies, ripasudil induced retraction and rounding as well as reduced actin bundles in monkey trabecular meshwork (TM) cells [27]. In addition, ripasudil reduced transendothelial electrical resistance (TEER), increased FITC-dextran permeability, and decreased ZO-1 immunostaining areas in monkey Schlemm's canal endothelial (SCE) cells [27]. These findings corroborate previous studies of other ROCK inhibitors in rabbits or monkeys [19, 20, 33–35]. Therefore, promotion of aqueous outflow by ripasudil is likely due to TM cytoskeletal changes, reduced outflow resistance, and increased SCE permeability as a result of ROCK inhibition. These results strongly indicate that the ocular hypotensive effect of ripasudil is associated with its potentiation of outflow facility from the conventional outflow route.

In this study, the IOP-lowering effect of ripasudil was enhanced by instillation with brimonidine, brinzolamide, latanoprost, latanoprost/timolol fixed combination, and dorzolamide/timolol fixed combination. Furthermore, combined instillation of ripasudil with latanoprost/timolol fixed combination showed more additive IOP-lowering effect compared with combined instillation of ripasudil with timolol or latanoprost. Therefore, additive IOP-lowering effect by ripasudil was able to show with two or more agents. These results suggest that increment of conventional outflow is effective for lowering the IOP under the increase in uveoscleral outflow or increment of uveoscleral flow with suppressing the aqueous humor production. However, combined instillation of ripasudil with nipradilol did not show additive effect on IOP compared with their single instillations. The maximum IOP-lowering effect of nipradilol was observed at 1 h after instillation, and the IOP value was 14.1 mmHg, which is similar to the episcleral venous pressure in rabbits [36]. Ripasudil and nipradilol have similar IOP-lowering effect, that is, both agents show maximum IOP reduction at 1 h after instillation and disappear rapidly in rabbits. This might be the reason why we could not take more additive IOP reduction by combination of ripasudil with nipradilol. On the other hand, combination of ripasudil with other agents prolonged the duration of IOP-lowering effects compared with single instillation of each agent. The ocular hypotensive mechanism of ripasudil, facilitation of conventional outflow, provides additive ocular hypotensive effect with combination use of other types of ocular hypotensive agents (Table 1). Furthermore, there was no adverse event regarding the topical instillation of ripasudil of coadministration of ripasudil with other antiglaucoma drugs in this study. Our results in this study agree with clinical studies, in that the administration of ripasudil with timolol or latanoprost showed additive IOP-lowering effect in glaucoma patients [31]. Therefore, we believe that the additive IOP-lowering effect of ripasudil with other glaucoma therapeutic agents in this study would provide beneficial clinical effects.

There are many reports that not only an elevation of IOP but also an impairment of ocular circulation are the etiology of glaucoma and evaluated the effect of antiglaucoma drugs on ocular blood flow in experimental animals and humans [37–42]. Nakabayashi et al. reported that ripasudil increased retinal blood flow in cats [43]. Similar results were reported by other ROCK inhibitor reagents [44, 45], and this effect might be due to direct vasodilating action of ROCK inhibitors in the posterior side of the eye.

Glaucoma is a condition that involves distinctive change in the optic nerve and visual field [23], and neuroprotective effect might be a beneficial effect on suppressing the progression of glaucomatous neural damage. There are many reports for the neuroprotective effect of antiglaucoma agents. Brimonidine showed neuroprotective effect in rats [46] and prevented the progression of visual field loss in humans [47]. Yamamoto et al. reported the neuroprotective effect of ripasudil in rats [48]; similar effects were also observed with other ROCK inhibitors [18, 49, 50]. Therefore, neuroprotective effect by ripasudil is expected to show beneficial effect on visual field in humans.

Furthermore, ROCK inhibitors have direct anti-inflammatory effects [51, 52] compared with other antiglaucoma agents. Increased production of proinflammatory cytokines has been reported to result in POAG and secondary, including exfoliation and uveitic glaucomas [53, 54]. Glucocorticoid-induced ocular hypertension is a form of secondary open-angle glaucoma induced by steroid administration. Its underlying mechanisms are associated with increasing outflow resistance through the conventional outflow route caused by accumulation of extracellular matrix (ECM) [55]. Fujimoto et al. reported that ROCK inhibitor improved dexamethasone-induced reduction of the outflow facility and inhibited the increase in ECM, such as collagen type IV *α*1 and fibronectin mRNA expression in porcine eyes [56]. Therefore, anti-inflammatory effect of ripasudil and its suppressive effect of ECM via ROCK inhibition may provide a strategy to treat and prevent secondary glaucoma, with additive IOP-lowering effects when combined regimens are used with other antiglaucoma agents.

## 5. Conclusions

In this study, we demonstrated that ripasudil showed additional maximum IOP-lowering effect or prolongation of IOP-lowering effect in combined regimens with *β*-blocker, *αβ*-blocker, *α*2-agonist, CAI, PG analog, and fixed combination of these agents. The mechanisms of action are due to increment of conventional outflow by ripasudil treatment. Ripasudil is expected to have substantial utility when used in combined regimens with existing agents and provide a greater choice in pharmacological treatment options for glaucoma.

## Figures and Tables

**Table 1:** Additive effect by ripasudil for categories of glaucoma therapeutic agents.

Categories	Target	Additive effect with ripasudil
*β*-Blockers	Suppression of aqueous humor production	IOP reduction Prolonged duration
CAI		IOP reduction Prolonged duration
Combination of *β*-blockers and CAI		IOP reduction Prolonged duration

PG analogs	Promotion of uveoscleral outflow	IOP reduction Prolonged duration

*α*2-Agonists	Suppression of aqueous humor production and promotion of uveoscleral outflow	IOP reduction Prolonged duration
*αβ*-Blockers		Prolonged duration
Combination of PG analogs and *β*-blockers		IOP reduction Prolonged duration
